# Protective Effects of Epigallocatechin Gallate (EGCG) on Endometrial, Breast, and Ovarian Cancers

**DOI:** 10.3390/biom10111481

**Published:** 2020-10-25

**Authors:** Yun-Ju Huang, Kai-Lee Wang, Hsin-Yuan Chen, Yi-Fen Chiang, Shih-Min Hsia

**Affiliations:** 1 School of Nutrition and Health Sciences, College of Nutrition, Taipei Medical University, Taipei 11031, Taiwan; d04641004@ntu.edu.tw (Y.-J.H.); hsin246@gmail.com (H.-Y.C.); yvonne840828@gmail.com (Y.-F.C.); 2 Department of Nursing, Ching Kuo Institute of Management and Health, Keelung 20301, Taiwan; kellywang@tmu.edu.tw; 3 Graduate Institute of Metabolism and Obesity Sciences, College of Nutrition, Taipei Medical University, Taipei 11031, Taiwan; 4 School of Food and Safety, Taipei Medical University, Taipei 11031, Taiwan; 5 Nutrition Research Center, Taipei Medical University Hospital, Taipei 11031, Taiwan

**Keywords:** epigallocatechin gallate, endometrial cancer, breast cancer, ovarian cancer, synergistic effect

## Abstract

Green tea and its major bioactive component, (−)-epigallocatechin gallate (EGCG), possess diverse biological properties, particularly antiproliferation, antimetastasis, and apoptosis induction. Many studies have widely investigated the anticancer and synergistic effects of EGCG due to the side effects of conventional cytotoxic agents. This review summarizes recent knowledge of underlying mechanisms of EGCG on protective roles for endometrial, breast, and ovarian cancers based on both in vitro and in vivo animal studies. EGCG has the ability to regulate many pathways, including the activation of nuclear factor erythroid 2-related factor 2 (Nrf2), inhibition of nuclear factor-κB (NF-κB), and protection against epithelial–mesenchymal transition (EMT). EGCG has also been found to interact with DNA methyltransferases (DNMTs) and histone deacetylases (HDACs), which affect epigenetic modifications. Finally, the action of EGCG may exert a suppressive effect on gynecological cancers and have beneficial effects on auxiliary therapies for known drugs. Thus, future clinical intervention studies with EGCG will be necessary to more and clear evidence for the benefit to these cancers.

## 1. Introduction

Green tea, derived from the *Camellia sinensis* plant, is the most popular beverage in East Asia. It has been used and consumed for health benefits in China since around 5000 years ago [1]. Catechins, active ingredients of green tea, contain (−)-epigallocatechin-3-gallate ((−)-EGCG), (−)-epigallocatechin ((−)-EGC), (−)-epicatechin-3-gallate ((−)-ECG), and (−)-epicatechin ((−)-EC) [2]. EGCG has various biological activities, including antioxidant, radical scavenging [3], antimicrobial, anti-inflammatory, anticarcinogenic, antiapoptotic, and metal-chelating activities [4,5,6]. Based on epidemiological data, EGCG offers potential protection from cancers related to hormones, such as breast or prostate cancers [7]. Current research revealed that EGCG with chemical modification, such as polymeric nanogels with matching small interfering RNA (siRNA), improved the sensitization involved with chemotherapy [8]. Taken together, the data indicated that treatment with EGCG or a combination of treatment and EGCG have synergistic anticancer effects, according to in vivo and in vitro studies [9].

Endometrial adenocarcinoma, breast cancer, and ovarian cancer are the most common invasive malignancies among women and the leading causes of death worldwide. In 1988, the International Federation of Gynecology and Obstetrics (FIGO) developed a pathological and surgical staging system for endometrial cancer [10]. Type I carcinomas commonly result from estrogen-driven obesity, whereas type II cancer is of serous papillary or clear-cell histology and has poor prognosis [11]. Recent studies have shown that the expression of DICER1 hotspot mutations promoted cell proliferation and aggression [12]; meanwhile, loss of the phosphatase and tensin homolog (PTEN), a tumor suppressor, is common in endometrial adenocarcinoma [13]. The overexpression of the p53 tumor-suppressor gene is positively correlated with gene mutation in advanced-stage (III or IV) endometrial cancers [14] and breast cancers [15]. A number of high-risk factors in endometrial cancers are excess exposure to estrogen, late onset of menopause, postmenopausal hormone replacement therapy, obesity, and inflammatory environments [16]. According to the clinical and pathological characteristics, a central strategy used total abdominal hysterectomy and bilateral salpingo-oophorectomy (TAH-BSO) and adjuvant radiation therapy to treat stage I endometrial carcinoma [10].

Breast cancer is a heterogeneous disease, and its distinctive biological subtypes include triple-negative phenotype (ER (estrogen receptor)−/PR (progesterone receptor)−/HER2 (human epidermal growth factor receptor 2)−) and hormone receptor-positive/human epidermal growth factor 2 (ERBB2; formerly HER2) ERBB2-negative and ERBB2-positive. Epidemiological evidence suggests that ovarian and other hormones are associated with the genetic or nongenetic risk factors dependent on tumor pathology [17]. Age at menarche or menopause, nulliparity, pregnancy history, lactation experience, and postmenopausal obesity are all risk factors of breast cancer [18]. Epidemiological studies suggest that cyclooxygenase-2 (COX-2), an inducible enzyme, plays an important role in the genesis of breast cancer in human tumor cell lines. A selective COX-2 inhibitor, celecoxib, at a dose of 150 to 1500 ppm/day, had a significant preventive effect of reducing tumor incidence and volume in an in vivo study [19]. A main strategy for the treatment of triple-negative breast cancer is conventional kinds of chemotherapy due to the absence of a target [20]. Patients who have hormone-receptor-positive tumors receive endocrine therapy, and those with ERBB2-positive cancer receive a monotherapy of an ERBB2-targeted antibody or in combination with chemotherapy [21].

It is suggested that the surface epithelial cells are the primary locus for ovarian cancer and divide into a number of different epithelial types, including serous, endometrioid, mucinous, and clear-cell carcinomas [22]. Some factors, such as incessant ovulation, hormonal stimulation, oncogenes, tumor-suppressor genes, growth factors, and chronic inflammation, contribute to the tumorigenesis or etiology of ovarian cancer [23]. However, it is difficult to identify the premalignant phase, resulting in the deadliest gynecological cancer [24]. Chemotherapy is still the first option for patients with ovarian cancer due to high sensitivity [25]. The reader is referred to the recent guidelines on endometrial adenocarcinoma, breast cancer, and ovarian cancer for more information about current medical diagnoses and treatments.

It is clear that the major challenge for chemotherapy is to develop greater effectiveness and less toxicity, because it lacks tumor specificity and has low safety margins [26]. Therefore, novel or alternative combination strategies are required. A number of anticancer studies have indicated that phytochemicals or bioactive compounds such as polyphenol and carotenoids have promising or adjuvant efficacy with few or mild side effects for gynecological cancer. Therefore, we present and summarize here the anticancer effects of EGCG on gynecological cancers for further study.

## 2. Cancer-Protective Mechanisms of EGCG

### 2.1. Antioxidant Activity

Green tea polyphenols possess an antioxidant capacity and pro-oxidant properties, both in vitro and in vivo. EGCG and other tea catechins have demonstrated mediated multiple mechanisms with the ability to scavenge reactive oxygen species (ROS) and modulate cell signaling [27,28]. When EGCG reacted with H_2_O_2_, the characteristics of the pro-oxidants generated hydrogen peroxide, the hydroxyl radical, and a superoxide anion. Studies have shown that green tea phenol increases levels of phase II antioxidant enzymes in rat livers, including glutathione peroxidase (GPx), reductase, glutathione-S-transferase (GST), catalase, quinone reductase, and superoxide dismutase (SOD), which are associated with the elimination of ROS [29]. The expression of phase II enzymes has been directly involved in the nuclear factor erythroid 2-related factor 2 (Nrf2) gene in mice [30]. Recently, it has been demonstrated that the activation of Nrf2 is highly related to tumorigenesis and the resistance of chemotherapeutics in type II endometrial cancer. Antioxidant response element (ARE) binding and the transcriptional activity of Nrf2 were increased by EGCG in human breast epithelial cells (MCF10A cells). Moreover, 100 µM of EGCG influenced the Nrf2 pathway, as well as its target protein heme oxygenase-1 (HO-1), in tamoxifen-resistant MCF-7 cells [31]. The upregulation of HO-1 and manganese superoxide dismutase (MnSOD) increased and activated AKT and extracellular signal-regulated kinases 1/2 (ERK1/2) after EGCG treatment [32]. For Nrf2 knockout mice, treatment with EGCG prevented oxidative damage, inflammation, and fibrosis but failed in the presence of Kelch-like ECH-associated protein (KEAP1) [33]. EGCG activated the Nrf2 signaling pathway to prevent hepatotoxicity induced by oxidative stress, according to in vivo and in vitro studies [34]. In a previous study, treatment with EGCG reversed the protein expression of Notch-1, resulting in decreased ROS production during Notch-1 silence in human umbilical vein endothelial cells (HUVEC) [35]. Jatuworapruk et al. [36] reported that EGCG increased the antioxidant capacity in healthy individuals. The upregulation of miR-210 is related to AKT, nuclear factor-κB (NF-κB), mitogen-activated protein (MAP) kinases, and cell cycle regulation by EGCG in A/J mice [27]. Polyphenols, specifically EGCG, have been shown to reduce cellular damage by scavenging ROS under conditions of high oxidative stress in cancer cells. Table 1 summarizes recent studies on the anticancer and potential molecular mechanisms of EGCG on endometrial, breast, and ovarian cancer cells in vitro, whereas animal studies are shown in Table 2.

### 2.2. Anti-Inflammatory Activity

**Figure 1:**
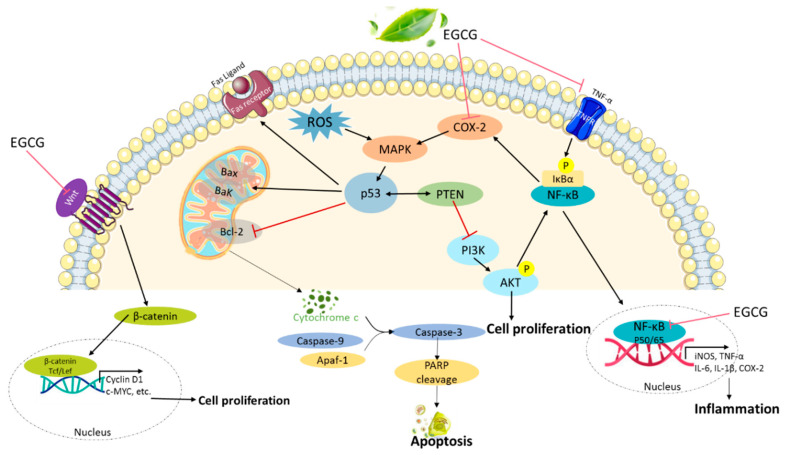
Molecular mechanisms of the (−)-epigallocatechin gallate (EGCG) inhibition of inflammation and proliferation and induction of apoptosis. EGCG downregulated multiple signal pathways, such as the PI3K/AKT, NF-κB, and Wnt signaling pathways, to inhibit cellular proliferation and inflammation and induce apoptosis. PI3K, phosphoinositide-3-kinase; AKT, protein kinase B; NF-κB, nuclear factor-κB; COX-2, cyclooxygenase-2; ROS, reactive oxygen species; and PTEN, phosphatase and tensin homolog.

Inflammation is a complex process that causes cellular and tissue damage by regulating inflammatory mediators such as nitric oxide (NO) and proinflammatory cytokines such as interleukins (IL-12, IL-1β, and IL-6) and tumor necrosis factor alpha (TNF-α). The primary cell of chronic inflammation is a macrophage activated by interferon-γ, proinflammatory cytokines, or bacterial lipopolysaccharides (LPS) [63]. Treatment with EGCG at doses of 10 μM and 50 μM showed inhibitory activity towards NO, COX-2, IL-6, IL-1β, and TNF-α in LPS-induced RAW 264.7 cells [63]. In addition, the neuroprotection of EGCG inhibited ROS production and attenuated inflammatory cytokines, including TNF-α, IL-1β, and IL-6, against LPS-mediated neurotoxicity in neuronal cultures [64]. It has also been suggested that EGCG inhibited the expression of Iba-1 and proinflammatory cytokines and ameliorated the IκB decrease in microglia of the rat hippocampus [65]. Moreover, pretreatment with EGCG effectively inhibited the PI3K/AKT/mTOR pathway in mesangial cells, but it was not involved in AMP-activated protein kinase (AMPK) activation [66]. The anti-inflammatory effect of EGCG at a dose of 50 mg/kg bw reduced TNF-α and IL-1β expression and improved histopathological results in the LPS-induced cell lines. Most of the in vitro studies showed that a high concentration of EGCG inhibited the proliferation of many different cancer cell lines. However, a high dose did not show a positive correlation with pharmacological activity [4,67]. In breast cancer cell lines, including MCF-7, T47D, MDA-MB-231, and HS578T, a low concentration at a dose of 25 μM of EGCG found a cytotoxic effect and synergistic cytotoxicity by a combination of EGCG and 4-hydroxytamoxifen (4-OHT) [39] or raloxifene [68] in MDA-MB-231 cells. In diabetic animals, EGCG 50-mg/kg administration downregulated the expression of Nrf2, heat shock protein 90 (HSP90), and HO-1 and increased the levels of serum cystatin C and neutrophil gelatinase-associated lipocalin (NGAL) as a prognostic role of renal damage [67]. Increased expression of SIRT1 and FOXO3a, two longevity factors, were recorded when the liver and kidney tissues of rats were treated with EGCG. These results suggest that the lifespan was prolonged by improving age-associated inflammation and oxidative stress [69]. The chemotherapeutic effects of EGCG significantly shrank tumors and reduced lipid peroxidation, leukocytosis, and C-reactive protein (CRP) as a predictor of tumor progression from cisplatin-induced nephrotoxicity [26]. Therefore, EGCG treatment with multiple cell lines reduced cell growth through a cytokinetic effect and suppressed ERK phosphorylation [70], epidermal growth factor receptor (EGFR), and the AKT signaling pathway [68] for multiple chemotherapeutics (Figure 1).

### 2.3. Antiproliferative Activity

Several studies have shown that NF-κB has an important role in tumorigenesis by regulating NF-κB target genes. These target genes are involved in proliferation, survival, inflammation, and metastasis as hallmarks of cancers [71]. NF-κB pathway activation is attenuated by EGCG through inhibiting the phosphorylation and subsequent degradation of the NF-κB repressor (IκBα), thereby blocking the nuclear translocation of p65 or p50 [57,72]. Several studies show that EGCG downregulated the NF-κB signaling pathway to inhibit proliferation and reduce the invasive potential [71,73,74]. Deregulated Wnt signaling remains an established model of breast tumorigenesis. G1 regulators, cyclin D1 and c-MYC, a Wnt target gene, overexpressed in breast cancers. By using DNA-based short hairpin RNA (shRNA) to knock down the HBP1 gene, EGCG was found to reduce the inhibition of Wnt signaling, promoting migration and invasiveness. Thus, treatment with EGCG reduced the invasiveness and proliferation by blocking Wnt signaling in an HBP1-dependent manner [40]. EGCG also inhibited tumor growth by blocking Wnt/β-catenin signaling, which depends on reduced glycogen synthase kinase-3β (GSK3β) activity in vivo [75,76]. β-Catenin, a transcription factor, regulates tumor cell proliferation in many cancers [75,77]. Cell proliferation markers such as Ki-67 and proliferating-cell nuclear antigen (PCNA) are associated with the inhibition of tumor growth. The Ki-67 protein is present in G1, S, G2, and mitosis, except G0, but the overexpression of PCNA is in the G1 and S phases of the cell cycle [78]. By increasing caspase-3, caspase-9, and poly (ADP-ribose) polymerase 1 (PARP-1), EGCG downregulated the expression of miR-25, which reduced the cell growth and proliferation rate, resulting in G2/M phase arrest and, ultimately, increased cell apoptosis in several breast cancer cell lines [41]. In clinical studies, free EGCG plasma levels are positively correlated with the change in Ki-67 after treatment with EGCG for four weeks in early breast cancer patients. This effect was found in the EGCG formulation with lecithin to improve absorption [79]. Estrogen plays an important role in influencing cell growth in breast cancer. In MCF-7 cells stimulated by estrogen, EGCG downregulated S-phase kinase protein 2 (Skp2) protein expression, an oncogenic that is overexpressed and correlated with the p27 protein decrease in breast cancer. In addition, p27 is a key regulator of G1-to-S-phase progression; thus, the level of p27 decreases in breast cancer as a poor prognosis [70]. EGCG combined with suberoylanilide hydroxamic acid (SAHA), a histone deacetylase (HDAC) inhibitor, affected the expression of miR-221/222, p27, and PTEN; it decreased for N-cadherin, whereas E-cadherin expression increased in the triple-negative breast cancer cells [42].

On the other hand, observation suggested that EGCG treatment arrested cells at the G1/S phase and facilitated apoptosis but did not influence CDK4 and Rb proteins in ovarian cancer cells [58]. It is also consistent with the results of the defective p53 status of SKOV3-ip1 and SKOV3TR-ip2 (paclitaxel-sensitive and -resistant) cells [80]. A clinical trial in which 118 patients with stage III or IV serous ovarian cancer were treated with indole-3-carbinol combined with EGCG at a dose of 200 mg resulted in prolonged progression-free survival and overall survival, as compared with that in untreated groups [81]. Besides, the high abnormal expression of aquaporin 5 (AQP5) is associated with tumorigenesis and the development of ovarian cancer by regulating the NF-κB pathway. Increased expression of PTEN and decreased AKT and mTOR were recorded when tumor cells of ovarian cancer xenograft-bearing nude mice were treated with 50-mg/kg EGCG [59]. Moreover, EGCG induced tumor-suppressor p53 phosphorylation and enhanced its transcriptional activity through the inhibition of MDM2-mediated p53 ubiquitination [82]. The treatment of Ishikawa cells with EGCG resulted in ERK signaling inhibition, including P38, JNK, c-jun, and c-fos, and proliferation marker downregulation of estrogen receptor α (ERα), progesterone receptor (PR), PCNA, and cyclin D1 [28]. Estradiol has been shown to act on proliferation and antiapoptosis in human endometrial cells. Estrogen receptor and progesterone receptors, ligand-activated transcription factors, are associated with hormone-mediated signaling and gene expression, cellular proliferation, and differentiation, as well as upregulation induced by estradiol treatment. When Ishikawa cells were incubated with EGCG and 17β-estradiol, they inhibited proliferation and cell cycle progression, showing an antiproliferative mechanism by AKT downstream MAPK, ERK, and c-jun N-terminal kinase (JNK) downregulation [37]. Recently published data showed that downregulated miR-381 expression not only directly targets IGF-1R but, also, indirectly regulates the ERK/AKT signal pathway in vitro [83].

### 2.4. Antiangiogenesis Effect

**Figure 2:**
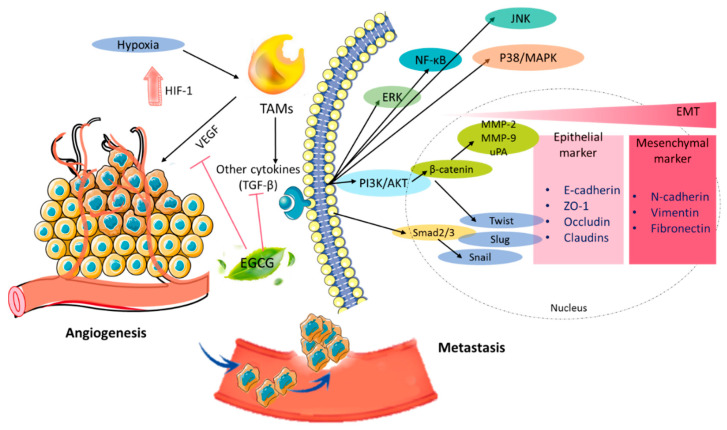
Molecular mechanisms of EGCG inhibition of angiogenesis and metastasis. EGCG downregulated the expressions of HIF-1 and multiple signal pathways, such as the PI3K/AKT, NF-κB, and MAPK signaling pathways, to inhibit angiogenesis and metastasis. PI3K, phosphoinositide-3-kinase; AKT, protein kinase B; HIF-1, hypoxia-inducible factor 1; VEGF, vascular endothelial growth factor; TAMs, tumor-associated macrophages; TGF-β, transforming growth factor-beta; and NF-κB, nuclear factor-κB.

Angiogenesis is a pivotal mediator of tumor progression, because tumor cell proliferation requires new blood vessel formation [84]. It is critical in developing the vasculature of a tumor for supporting its metabolic demands to tumor progression. Increased vascular endothelial growth factor (VEGF) stimulated by hypoxia-inducible factor (HIF-1) are involved in angiogenesis [85]. Tumor-associated macrophages (TAMs) have pro- and anti-inflammatory properties and serve the malignancy by enhancing immunosuppression, hem-/lymphangiogenesis, invasion, and metastasis as angiogenic factors. The presence of TAMs has been reported to be correlated with poor prognosis in tumors, including breast, ovary, and endometrial carcinomas [11]. EGCG decreased the expression of HIF-1α and VEGF in a dose-dependent manner in MCF-7 cells, indicating that EGCG inhibits cell growth and proliferation [43]. Moreover, its cells also found that EGCG significantly inhibited the activation of HIF-1α and VEGF expression through blocking NF-κB activation in E0771 cells [44]. In clinical studies, it has also been shown that EGCG significantly reduced VEGF secretion for the inhibition of angiogenesis [85]. On the other hand, adrenomedullin was found to be inhibited by EGCG that reduced VEGF release in endometrial cancer cells [86]. An in vivo study has suggested that EGCG inhibited angiogenesis and induced regression of the endometriotic lesions but did not influence ovarian follicles (Figure 2) [61].

### 2.5. Antimetastatic Activity

The poor prognosis of cancer is related to invasiveness and metastasis, which is the switch of tumor cells from an epithelial to epithelial-to-mesenchymal transition (EMT) phenotype and the related processes. EMT downregulated the expression of surface E-cadherin, resulting in the loss of homotypic adhesion, and upregulated Zeb-1, Zeb-2, and Snail as transcriptional repressors. In particular, the increase of transforming growth factor-beta (TGF-β) represents the progression of a solid tumor. TGF-β downregulates miR200 family microRNAs by many mechanisms, including the accumulation of Zeb-1 and Zeb-2, suppression of E-cadherin expression, and increased motility and invasiveness [87,88]. Several studies have indicated that EGCG appeared to be an inhibitor of cancer cell metastasis via an increase in the expression of E-cadherin and inhibition of the expression of N-cadherin, Zeb-1, MMP-2, and MMP-7 in tumor tissues [89]. MMP-2, MMP-7, MMP-9, and MMP-12 belong to metalloproteinases (MMPs), a family of zinc-binding enzymes related to tumor invasion and angiogenesis [90]. By treating MCF-7 cells or MDA-MB-231 with EGCG, the expression of type-1-matrix metalloproteinase (MT1-MMP), focal adhesion kinase (FAK) downregulation, and the adhesion of breast cells to fibronectin and vitronectin reduced in a time-dependent manner, indicating the inhibition of the process of pro-MMP-2 activity [45,46]. According to another study, EGCG decreased MMP-2 expression through JNK signaling [91] and inhibited MMP-7, MMP-9, and MMP-12 in tumor tissues [92]. Furthermore, the levels of urokinase-type plasminogen activator (uPA) play a major role in tumor invasiveness and metastasis as a strong prognostic factor [93,94]. EGCG also suppressed uPA activities and the inhibition of the AKT and ERK pathways to facilitate anti-invasion effects [94]. Among them, EGCG mediated tumor proteinase and the locking of the endothelin A receptor/endothlelin-1 (ET_A_R/ET-1) autocrine signaling pathway in ET-1-stimulated HEY cells. The ET_A_R-driven downstream signaling pathway includes the MAPK and PI3K pathways [60]. It has been reported previously that overexpression of the ET-1/ET _A_R autocrine loop correlates with primary and metastatic ovarian progression. The PI3K/AKT signaling pathway is implicated for transducing extracellular signaling and, in control cell–cell junctions, cell polarization, enhanced motility, and cell-matrix adhesion in the EMT. According to in vitro and in vivo studies, treatment with EGCG inhibits PI3K/AKT protein expression in breast cancer [47,48,95]. Altogether, the above studies may suggest that EGCG has the potential to target multiple signaling pathways, including VEGF receptor (VEGFR), MAPKs, PI3K/AKT, and protein kinase C (PKC) pathways, to suppress cell metastasis.

### 2.6. Induction of Apoptosis

The chemopreventive properties of EGCG have been widely reported to induce apoptosis by AMPK activation and tumor promotion enzymes such as COX-2 inhibition. Numerous in vitro and in vivo studies have looked at downregulating COX-2-derived prostaglandin synthesis [96]. Survival gene expression, such as VEGF and glucose transporter 1 (GLUT-1), is related to the chemoresistance process. EGCG has shown that the induction of cell death by increasing apoptosis-related proteins, such as caspase-3, caspase8, caspase9, Fas, cytochrome c, PTEN, bad, and smac, suppressed the antiapoptotic proteins B-cell leukemia/lymphoma-2 protein (Bcl-2), bcl-x_L_, and c-myc in 4T1 breast cancer cells [49]. Three silenced targets of hormonal cancer therapy of triple-negative breast cancer (TNBC) include ERα, progesterone receptor, and human epidermal growth factor receptor 2 (HER2). EGCG acts as an epigenetic alteration in changing the expression of the cellular inhibitor of apoptosis 2 (cIAP2) by histone modification. EGCG significantly reduced the epigenome-modifying enzymes of histone deacetylases (HDACs) in three TNBC cell lines and increased the proapoptotic caspase 7 expression [50]. Therefore, other studies investigating EGCG found epigenetic effects for reducing the metastatic potential of breast cancer. Likewise, EGCG treatment of human endometrial cancer cells (Ishikawa cells) resulted in the suppression of antiapoptotic protein Bcl2, the upregulation of proapoptotic Bax, and the activation of caspase-3 and poly (ADP-ribose) polymerase, the hallmark of apoptosis [28,37]. Moreover, EGCG induced ROS generation, which may be attributed to one of the mechanisms inducing apoptosis in endometrial carcinoma cells [28]. These effects of induction apoptosis are also found with the treatment of 40-µg/mL EGCG in SKOV3 cells of ovarian cancer cell lines [59]. Similar results have been reported on potential apoptosis induction in SKOV3TR-ip2 (paclitaxel-resistant) cells [80] or MCF-7 and MDA-MB-231 cells [97] treated with EGCG by decreasing human telomerase reverse transcriptase (hTERT) and Bcl-2 expression. The treatment with EGCG was also found to induce apoptosis and decrease the levels of p-HER2, AKT, and ERK1/2 proteins in SK-Br3 breast cancer cells. Furthermore, EGCG had a specific inhibition of the activity of the enzyme fatty acid synthase (FASN). Overexpression of FASN is common in breast cancer and human carcinomas [51]. EGCG may affect not only lipogenesis but, also, glucose metabolism, especially in glycolysis such as hypoxia-inducible factor 1α (HIF1α) and GLUT1, according to an in vivo study [49]. Treatment with EGCG promoted the expression of p53 to inhibit Bcl-2 for the induction of apoptosis in silent P53-transfected MCF-7 cells [44,52]. It suggests that EGCG downregulated miR-25, resulting in induced apoptosis in MCF-7 cells [41]. Recently, miR-34a, the target gene of p53, inhibits p53-dependent apoptosis by deacetylating in p53 acetylation sites. The upregulation of the miR-34a/Sirt1/p53 signaling pathway results in cancer cell migration and invasion. In H22 cells, EGCG inhibited the miR-34a/Sirt1/p53 signaling pathway to decrease the expression of Bcl-2 for radiation protection [98].

Additional studies indicate that EGCG downregulates telomerase activity associated with the inhibited expression of hTERT, a catalytic subunit of telomerase [99,100], because tumors with short telomeres caused chromosomal damage, resulting in triggering apoptotic cell death [101]. Moreover, pretreatment with EGCG in tumor necrosis factor (TNF)-related apoptosis-inducing ligand (TRAIL)-mediated cell modulated intrinsic, as well as the extrinsic, apoptotic pathways. It demonstrated synergistic inhibition of the angiopoietins and uPA, which is involved in angiogenesis and metastasis [102]. In addition, EGCG-treated cells reduced J-aggregates of mitochondrial membrane depolarization and released cytochrome c from the mitochondria to the cytosol. Mitochondrial membrane depolarization appears at the early stage of apoptosis due to permeability transition [103]. These studies concluded that EGCG can act on the induction of apoptosis by multiple mechanisms as cancer chemoprevention.

### 2.7. Autophagy and Exosome

Autophagy is a conserved catabolic process that is involved in cell growth, survival, and cell death [104]. Previous studies compared the differences in the methylation of autophagy genes such as Atg5 and LC3B in macrophages between young and aged mice. The hypermethylation of LC3 and Atg5, autophagosomal markers, is associated with the expression of high-level DNA methyltransferase-2 (DNMT2) in aged macrophages, resulting in a low expression of autophagy molecules [62]. In 4T1 cells of breast cancer, with 40-µM EGCG, the formation of autophagosomes fusing with the lysosomes was observed and increased the levels of Beclin1, Atg5, and LC3B-II/LC3B-I, resulting in cytosolic acidification and induction autophagy [49]. According to an in vivo study, EGCG induced autophagy to form autophagosomes and increased the phosphorylation of AMPK in the cell lines [105]. Cotreatment with EGCG plus p53 siRNAs also had a synergistic effect on the induction of autophagy in the Hs578T cell culture model of triple-negative breast cancer [53]. Using EGCG to reduce the methylation activity of DNA methyltransferase (DNMT), it was found that EGCG restored ATG5 and LC3 expression in aged macrophages, resulting in improved autophagy in senescence [62].

### 2.8. Bioavailability

EGCG showed a higher concentration in the digestive system, but the issue of its bioavailability resulted from its poor absorption, instability under neutral or alkaline conditions, and biologically inactivating processes such as methylation [106,107]. The methylation of EGCG by catechol-O-methyltransferase (COMT) was less bio-effective in breast cancer MDA-MB-231 cells [108]. Nanoparticles of EGCG, including folic acid, and polyethylene glycol treatment upregulated PTEN, p21, and Bax and downregulated p-PDK1, p-AKT, cyclin D1, and Bcl-2 in MCF-7 cells [54]. Pro-EGCG such as octaacetate [31] or peracetate-protected EGCG suppressed xenograft tumor growth, inhibited tumor angiogenesis, and reduced the expression of vascular endothelial growth factor A (VEGFA) and HIF1α by the PI3K/AKT/mTOR signaling pathway [38]. To improve the anticancer ability, conjugated EGCG with dual drug-loaded polystyrene–polysoyaoil–diethanol amine nanoparticles (PS–PSyox–NPs) [109] or loaded nanostructured lipid carriers–arginyl-glycyl-aspartic acid (NLC–RGDs) [110] effectively inhibited tumor growth and reduced toxicity, according to an in vitro breast cell line study. On the other hand, using chitosan-coated silica with EGCG to treat SKOV-3 cell lines improved cancer-targeted drug delivery by decreasing the hTERT, as a target of cancer therapy, and ERK2 [111]. Therefore, EGCG has the ability to enhance the drug-delivery efficiency to target sites as receptor-targeting groups.

### 2.9. Pharmaceutical Synergistic Effect

Chemotherapy is still one of the routine methods for cancer therapy, but it has severe side effects, drug resistance, low effectiveness, and a lack of selectivity. Therefore, new combination strategies for cancer therapy have been developed. EGCG has cancer chemopreventive properties due to attacking various targets in transformed cells. Cisplatin (cis-diamminedichloroplatinum, cDDP) is a major chemotherapeutic agent for the treatment of many types of cancer, including ovarian cancer. The cytotoxic effects of cDDP induced cell death by DNA damage, and its resistance resulted from multiple mechanisms. Recent studies suggested that copper transporters mediate the cellular pharmacology and sensitivity to platinum-based agents. Thus, EGCG induced copper transporter 1 (CTR1) protein expression and enhanced the sensitivity of ovarian cancer (OVCAR3 and SKOV3) cells to cDDP [112]. Another study showed that EGCG also increased the toxicity of cisplatin and accentuated oxidative stress [113]. A combination of trans-palladiums with EGCG exhibited a synergistic effect in the accumulation of platinum and the level of platinum−DNA binding [114]. In breast cancer studies with decitabine or 5-aza-2′-deoxycytidine (5-aza 2′dC), inhibition of the activity of DNA methyltransferase enzymes is mediated in cell cycle arrest and apoptosis by altering methylated apoptotic genes. Cotreatment with 5-aza 2′dC and EGCG significantly altered the tumorigenicity of the MCF-7 cell, including the inhibition of cell growth, change of the cell cycle, downregulation of DNA methylation, and histone modifications [115]. Additionally, raloxifene, a selective estrogen receptor modulator, induces apoptosis in a variety of cancer cells, such as ERα+ and ERα− breast cancer cells. Thus, the combination of raloxifene and EGCG elicited synergistic cytotoxicity by decreasing the phosphorylation of EGFR and AKT protein expression and induction of apoptosis earlier [68] (Table 3).

## 3. Conclusions

The health benefits associated with green tea consumption have been demonstrated in anticancer properties, which are attributed to the polyphenolic compounds present in green tea, particularly EGCG. Here, we reviewed the anticancer effects of EGCG in vitro and in vivo and focused on the potential molecular targets of endometrial, breast, and ovarian cancers. Additionally, encapsulating EGCG into nanoparticles increases its stability and improves the efficiency of the anticancer activity. However, limited information is available of clinical trials to show or support the suppressive effects of EGCG on endometrial, breast, and ovarian cancers. The failure of current therapies due to the existence of cancer stem cells caused cancer recurrence and progression. Therefore, the potential beneficial effects of EGCG on cancer, EGCG combined with conventional cytotoxic drugs, or a novel target therapy need further investigation in terms of clinical research. Overall, EGCG is a promising candidate for a natural product with therapeutic effects in endometrial, breast, and ovarian cancers.

## Figures and Tables

**Table 1:** Anticancer effects of epigallocatechin gallate (EGCG) based on cell studies.

Cancers	Cell lines	EGCG Concentrations	Anticancer Actions	Potential Molecular Mechanisms
Endometrial cancer	HEK-293 and Ishikawa cells	100, 125, and 150 µM	Inhibit cell proliferation and induce apoptosis	Activation of the P38 MAP kinase [28]
	Ishikawa cells	100 μM	Inhibit cell proliferation and induces apoptosis	Inhibition of the AKT and MAPK signal pathways [37]
	AN3CA, RL95-2, THP-1, and PHES	Pro-(peracetate) 20, 40, and 60 µM	Inhibit tumor angiogenesis	Downregulation of HIF1α/VEGFA through the PI3K/AKT/mTOR/HIF1α pathway [38]
Breast cancer	MCF-7	100 μM	Antioxidant Induce apoptosis	Inhibition of the Nrf2 signaling pathway [31]
	MCF-7, T47D, MDA-MB-231, and HS578T	5–25 μM		Evaluation of cytotoxicity [39]
	MDA-MB-231	25–100 μM	Inhibit invasion	Inhibition of Wnt signaling and target gene c-MYC [40]
	MCF-7	5 and 20 μg/mL	Induce apoptosis	Upregulation of caspase 3, caspase 9, and PARP [41]
	MCF-7, MDA-MB-157, MDA-MB-231, and HCC1806	5 μM	Inhibit migration	Inhibition of N-cadherin and increase of E-cadherin [42]
	MCF-7	25, 50, and 100 mg/L	Inhibit proliferation	Downregulation of HIF-1α and VEGF [43]
	E0771, MCF-7, and MDA-MB-231	10, 20, and 50 ug/mL	Inhibit proliferation and migration	Downregulation of HIF-1α and NF-κB [44]
	MCF-7	5, 10, and 20 μM	Inhibit invasion	Downregulation of the PI3K/ERK/NF-κB pathway [45]
	MDA-MB-231	20 μM	Inhibit invasion	Downregulation of the FAK/ERK/NF-κB pathway [46]
	MDA-MB-231	10 and 20 μM	Inhibit invasion	Downregulation of the FAK/PI3K/AKT pathway [47]
	T47D	0–80 μM	Induce apoptosis	Downregulation of the PI3K/AKT pathway [48]
	4T1	10–320 μM	Induce apoptosis	Upregulation of caspase 3, caspase 8, and caspase 9 [49]
	MCF-7, MDA-MB-157, MDA-MB-231, and HCC1806	5 μM	Inhibit metastasis	Increase proapoptotic caspase 7 [50]
	MCF-7, MDA-MB-231, and SK-Br3	20–150 μM	Induce apoptosis	Inhibition of FASN activity and downregulation of the ERK/AKT pathway [51]
	MCF-7	20~120 μmol/L	Induce apoptosis	Downregulation of the P53/Bcl-2 signaling pathway [52]
	Hs578T	40 nmol	Induce apoptosis Activation autophagy	Target apoptotic and angiogenic pathways [53]
	MCF-7	Nanoparticle 200 μg/mL	Inhibit proliferation	Regulation of the PI3K-Akt pathway [54]
	MCF-7	10 μM	Inhibit proliferation	Downregulation of Skp2 [55]
	DMBA-transformed human D3–1	60 μg/mL	Inhibit angiopoietin	Alteration expression related to nuclear and cytoplasmic transport, transformation, and redox signaling [56]
Ovarian cancer	SKOV3	20–100 μg/ mL	Inhibit proliferation and induce apoptosis	Downregulation of AQP5, NF-κB, p65, and IκB-α [57]
	SKOV-3, OVCAR-3, and PA-1	25, 50, and 100 µM	Induce apoptosis	Upregulation of P21 and Bax and downregulation of BCL-X_L_ and PCNA [58]
	SKOV3, CAOV-3, and NIH-OVCAR-3	5, 10, 20, 40, and 80 µg/mL	Inhibit proliferation and induce apoptosis	Upregulation of Bax and caspase-3 and downregulation of Bcl-2 [59]
	HEY and OVCA 433	20–40 μmol/L	Inhibit proliferation and induce apoptosis	Downregulation of ET_A_R-dependent signaling pathways [60]

Abbreviations: AN3CA, metastatic undifferentiated EC; THP-1, the human leukemia cell line; MAPK, mitogen-activated protein kinase; AKT, protein kinase B; HIF1α, hypoxia-inducible factor 1α; VEGFA, vascular endothelial growth factor A; PI3K, phosphatidylinositol 3-kinase; mTOR, mammalian target of rapamycin; Nrf2, nuclear factor erythroid 2-related factor 2; PARP-1, Poly(ADP-ribose) polymerase 1; VEGF, vascular endothelial growth factor; HIF-1α, hypoxia-inducible factor-1α; NF-κB, nuclear factor-κB; ERK, extracellular signal-regulated kinase; FAK, adhesion-mediated focal adhesion kinase; FASN, fatty acid synthase; Skp2, S-phase kinase protein 2; IκBα, inhibitor of nuclear factor kappa B; BCL-XL, B-cell lymphoma extra-large; PCNA, proliferating-cell nuclear antigen; ETAR, selective receptor ETA.

**Table 2:** Anticancer effects of EGCG based on animal studies.

Cancers	Animal models	EGCG treatments	Potential molecular mechanisms
Endometrial cancer	Transgenic luciferase-expressing mice (CMV-Luc)	EGCG and Pro-(EGCG octaacetate) 50 mg/kg	Inhibits tumor growth and angiogenesis [31]
	Female Syrian golden hamsters	65 mg/kg	Inhibits VEGF expression [61]
Breast cancer	Female C57BL/6 mice	50-100 mg/kg	Inhibits tumor VEGF expression [44]
	C57BL/6 aged (62-64 weeks old) and young (8 weeks old) mice	300 μg/30 μl DMSO	Inhibits DNA methyltransferase 2 (DNMT2) methylation activity [62]
Ovarian cancer	Female BALB/c nude mice	50 mg/kg	Inhibits tumor growth by regulating the PTEN/AKT/mTOR pathway [59]
	Female athymic (nu+/nu+) mice	12.4 g/L	Inhibition of tumor growth by the reduction of ET_A_R and ET-1 expression [60]

Abbreviations: VEGF, vascular endothelial growth factor; PTEN, phosphatase and tensin homolog; AKT, protein kinase B; mTOR, mammalian target of rapamycin; ETAR, selective receptor ETA; ET-1, the endothelin-1.

**Table 3:** Pharmaceutical synergistic effects of EGCG.

Cancer	Cell Lines	EGCG Treatments	Drugs	Cytotoxic Action
Breast cancer	MDA-MB-231	25 µM	raloxifene	Induce apoptosis [68]
	MCF-7 and MDA-MB 231	50 µM	5-aza 2′dC	Changes in DNA methylation and histone modifications [115]
Ovarian cancer	SKOV3-ip1 and SKOV3TR-ip2 (paclitaxel-sensitive and -resistant)	5, 10, 20, and 30 µM	Paclitaxel	Induce apoptosis by the downregulation of Bcl-2 [80]
	OVCAR3, SKOV3, and HEK-293T cells	10 μM	Cisplatin	Upregulation of CTR1 and increase cDDP accumulation [112]
	CAOV3, SKOV3, OVCAR3, OVCAR10, A2780, CP70, C30, and C200	6.3, 12, 25, and 50 µM	Cisplatin	Increase in intracellular oxidative stress [113]
	A2780 (cisplatin-sensitive, parental cell line), A2780cisR (cisplatin-resistant), and A2780ZD0473R (ZD0473-resistant)	1.37–21.98 μM and 1.33–21.34 μM	Cisplatin	Accumulation of platinum and a level of platinum−DNA binding [114]

## Abbreviations

EGCG	(−)-epigallocatechin gallate
Nrf2	nuclear factor erythroid 2-related factor 2
NF-Κb	nuclear factor-κB
EMT	epithelial–mesenchymal transition
DNMTs	DNA methyltransferases
HDACs	histone deacetylases
EGC	(−)-epigallocatechin
ECG	(−)-epicatechin-3-gallate
EC	(−)-epicatechin
siRNA	small interfering RNA
FIGO	Federation of Gynecology and Obstetrics
PTEN	phosphatase and tensin homolog
TAH-BSO	total abdominal hysterectomy and bilateral salpingo-oophorectomy
ER	estrogen receptor
PR	progesterone receptor
HER2	human epidermal growth factor receptor 2
COX-2	cyclooxygenase-2
ROS	reactive oxygen species
GPx	glutathione peroxidase
GST	glutathione-S-transferase
SOD	superoxide dismutase
ARE	antioxidant response element
HO-1	heme oxygenase-1
MnSOD	manganese superoxide dismutase
ERK1/2	extracellular signal-regulated kinases ½
KEAP1	Kelch-like ECH-associated protein
HUVEC	human umbilical vein endothelial cells
MAP	mitogen-activated protein
NO	nitric oxide
TNF-α	tumor necrosis factor alpha
LPS	lipopolysaccharide
AMPK	AMP-activated protein kinase
4-OHT	4-hydroxytamoxifen
HSP90	heat shock protein 90
NGAL	neutrophil gelatinase-associated lipocalin
CRP	C-reactive protein
EGFR	epidermal growth factor receptor
shRNA	short hairpin RNA
GSK3β	glycogen synthase kinase-3β
PCNA	proliferating-cell nuclear antigen
PARP-1	poly (ADP-ribose) polymerase 1
Skp2	S-phase kinase protein 2
SAHA	suberoylanilide hydroxamic acid
AQP5	abnormal expression of aquaporin 5
ERα	estrogen receptor α
VEGF	vascular endothelial growth factor
HIF-1	hypoxia-inducible factor
TAMs	tumor-associated macrophages
TGF-β	transforming growth factor-beta
MMPs	metalloproteinases
MT1-MMP	type-1-matrix metalloproteinase
FAK	focal adhesion kinase
JNK	c-jun N-terminal kinase
uPA	urokinase-type plasminogen activator
ETAR/ET-1	endothelin A receptor/endothlelin-1
PKC	protein kinase C
GLUT-1	glucose transporter 1
TNBC	triple-negative breast cancer
cIAP2	cellular inhibitor of apoptosis 2
hTERT	human telomerase reverse transcriptase
FASN	fatty acid synthase
HIF1α	hypoxia-inducible factor 1α
MDR1	multidrug resistance 1
TRAIL	tumor necrosis factor (TNF)-related apoptosis-inducing ligand
DNMT2	DNA methyltransferase-2
DNMT	DNA methyltransferase
COMT	catechol-O-methyltransferase
VEGFA	vascular endothelial growth factor A
PS–PSyox–NPs	polystyrene–polysoyaoil–diethanol amine nanoparticles
NLC-RGDs	nanostructured lipid carriers–arginyl-glycyl-aspartic acid
CTR1	copper transporter 1
5-aza 2′dC	decitabine or 5-aza-2′-deoxycytidine
